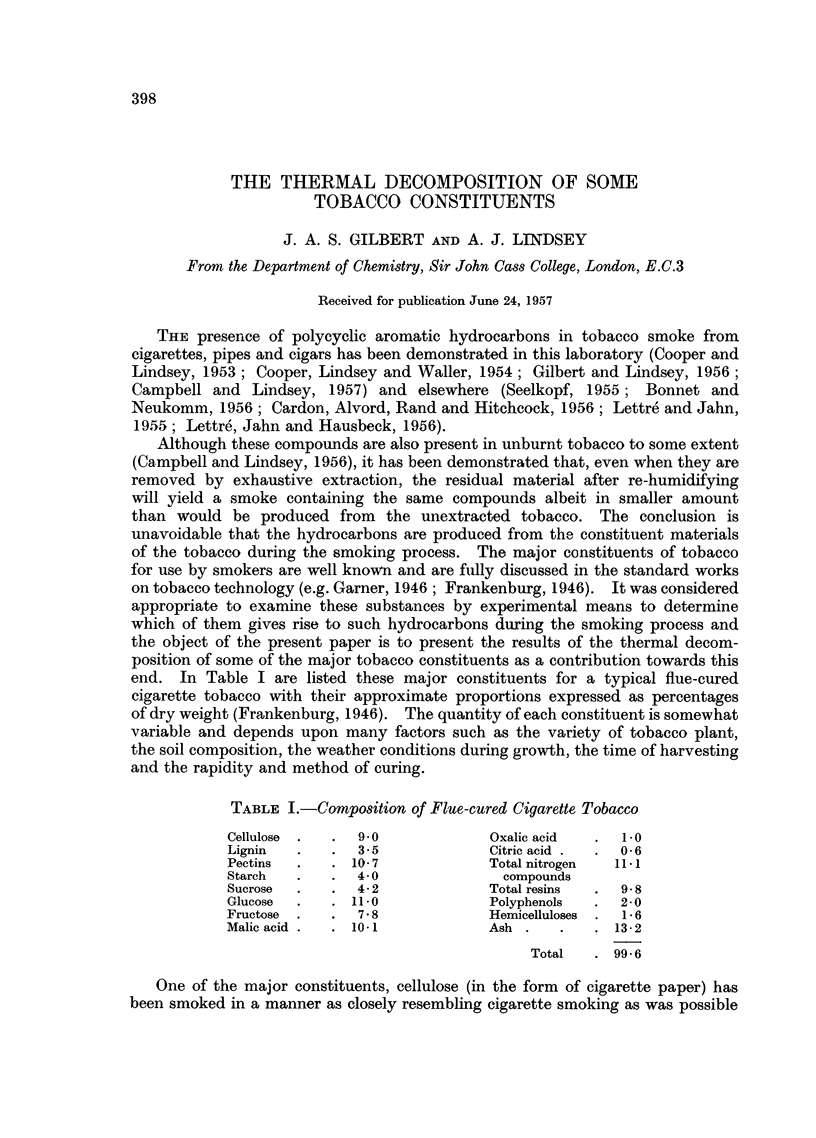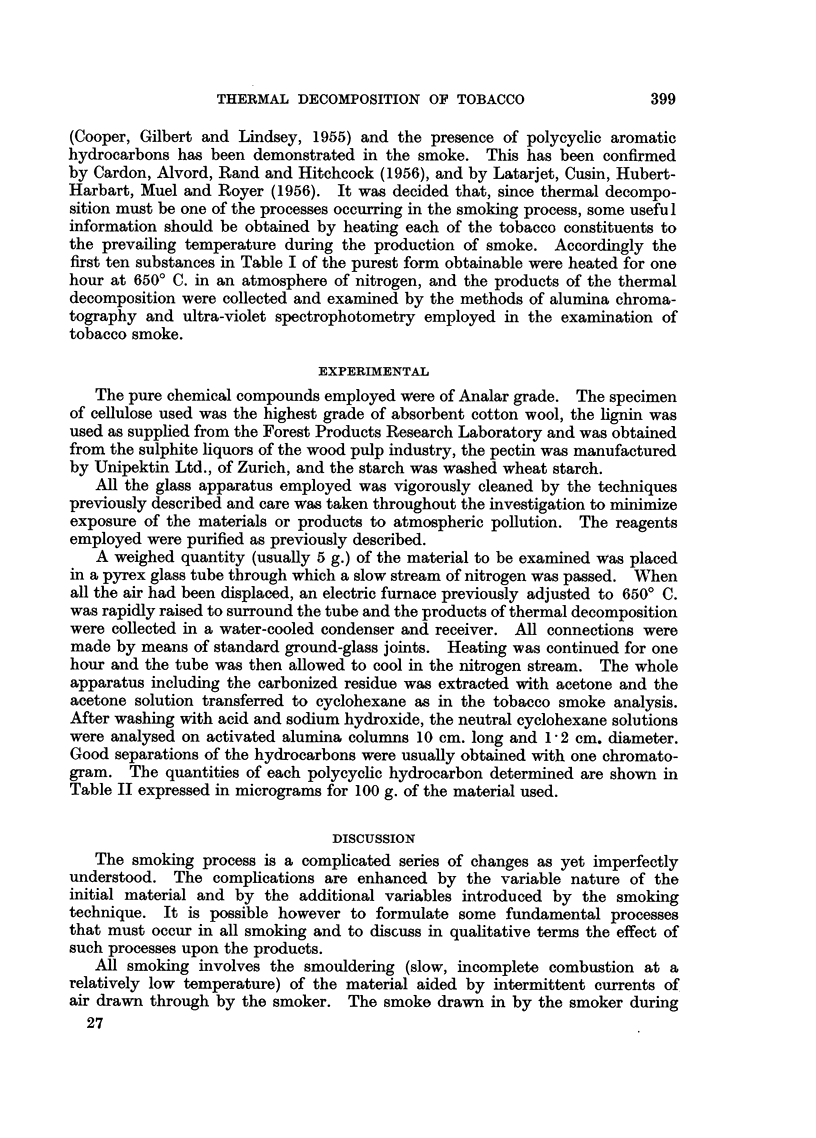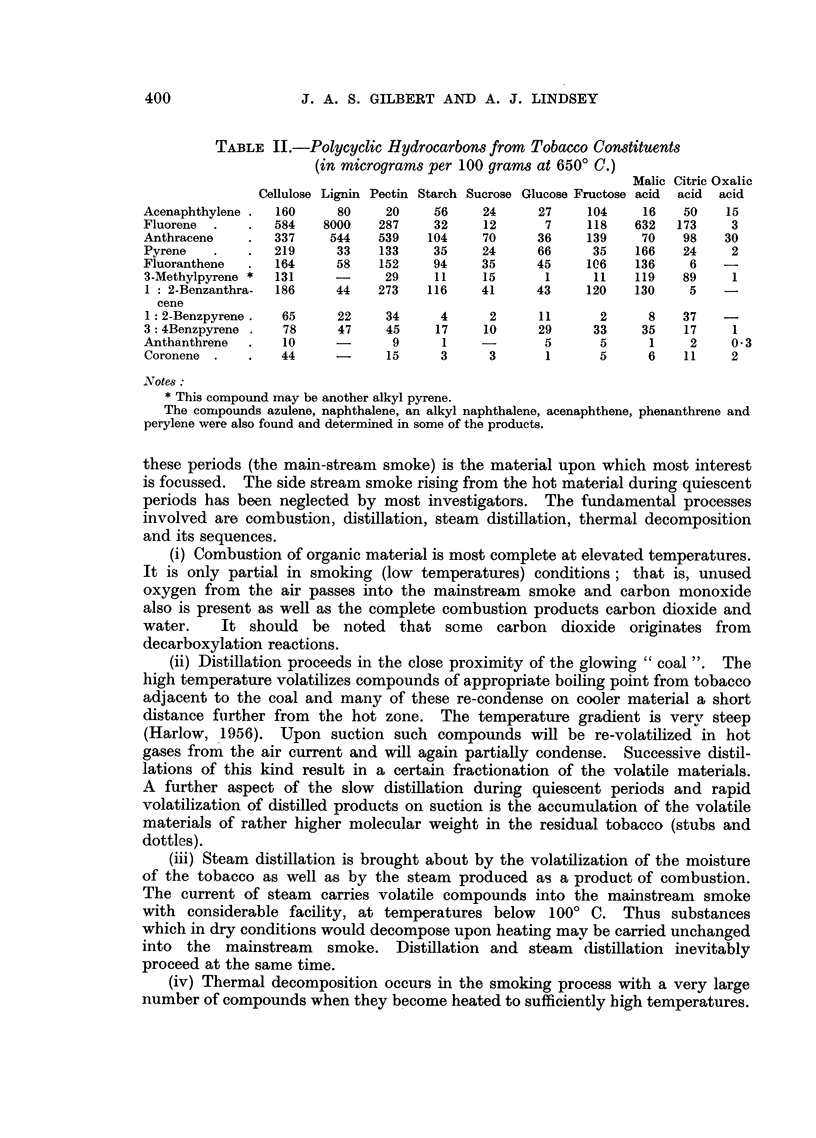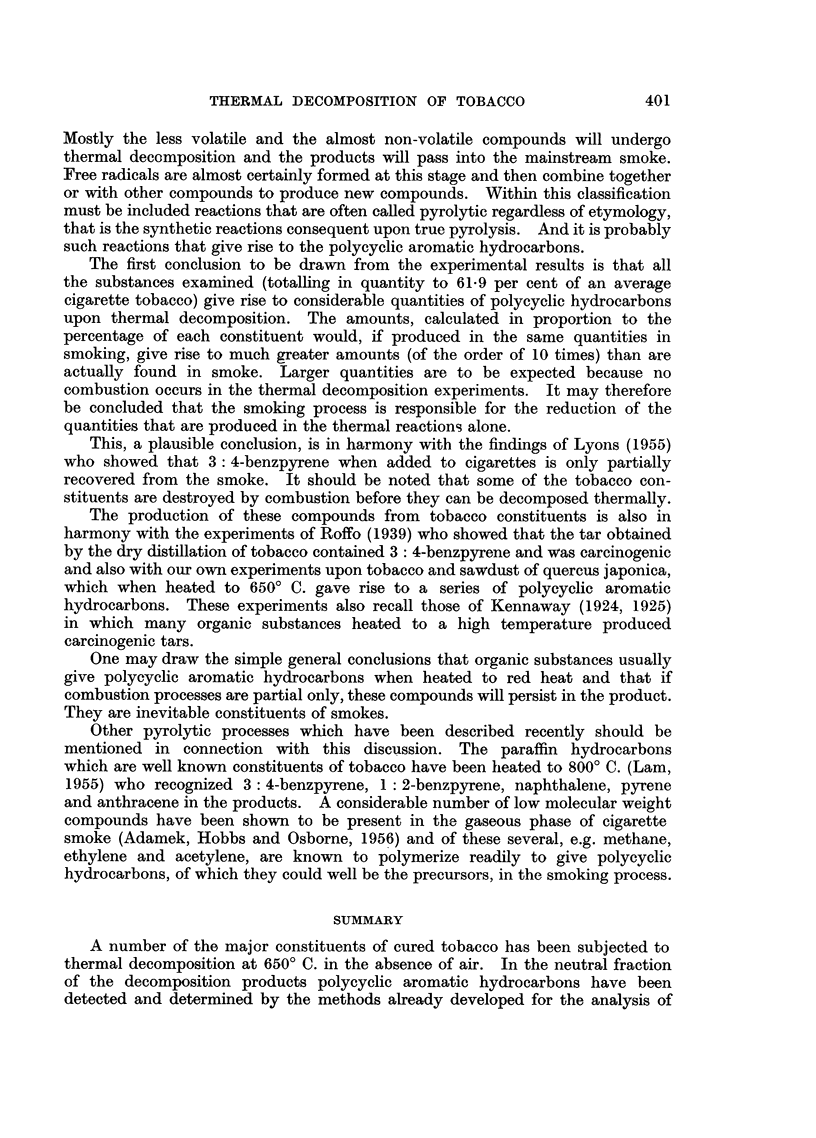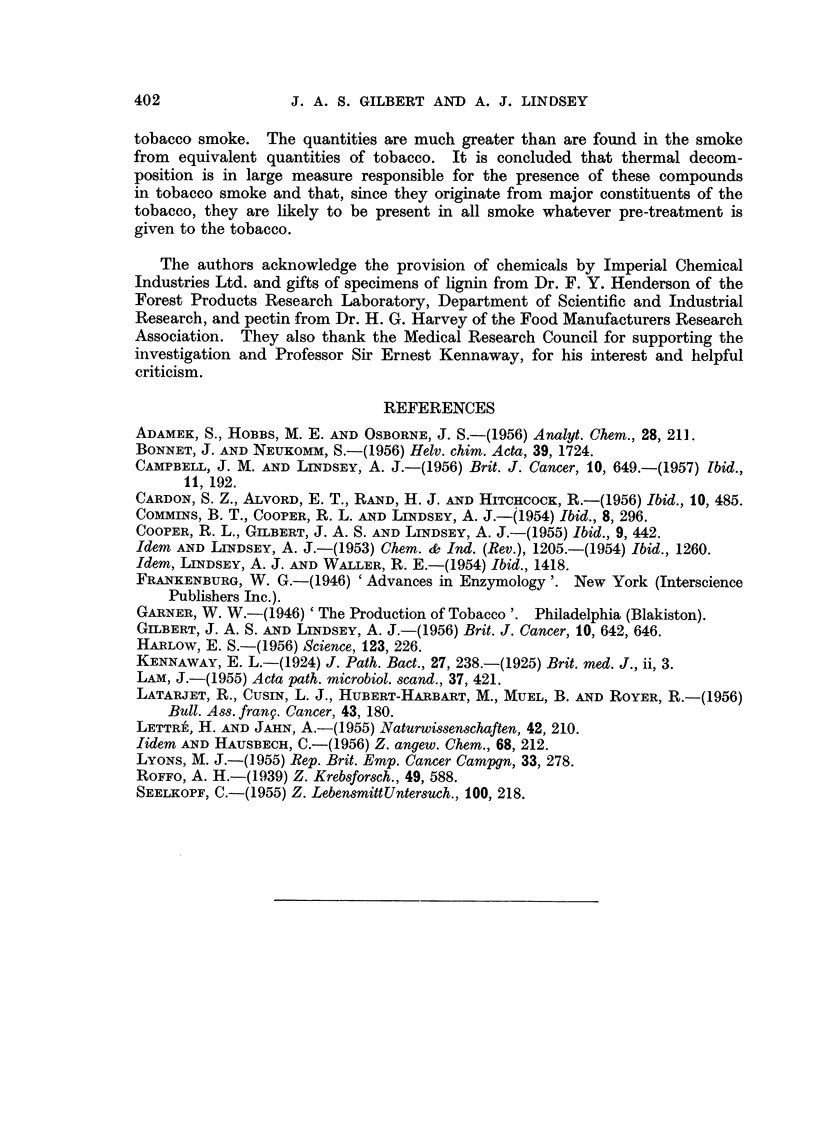# The Thermal Decomposition of Some Tobacco Constituents

**DOI:** 10.1038/bjc.1957.47

**Published:** 1957-09

**Authors:** J. A. S. Gilbert, A. J. Lindsey


### THE THERMAL DECOMPOSITION OF SOME TOBACCO CONSTITUENTS

J. A. S. GILBERT AND A. J. LINDSEY

*From the Department of Chemistry, Sir John Cass College, London, E.C.3*

Received for publication June 24, 1957

THE presence of polycyclic aromatic hydrocarbons in tobacco smoke from cigarettes, pipes and cigars has been demonstrated in this laboratory (Cooper and Lindsey, 1953 ; Cooper, Lindsey and Waller, 1954 ; Gilbert and Lindsey, 1956 ; Campbell and Lindsey, 1957) and elsewhere (Seelkopf, 1955 ; Bonnet and Neukomm, 1956 ; Cardon, Alvord, Rand and Hitchcock, 1956 ; Lettré and Jahn, 1955 ; Lettré, Jahn and Hausbeck, 1956).

Although these compounds are also present in unburnt tobacco to some extent (Campbell and Lindsey, 1956), it has been demonstrated that, even when they are removed by exhaustive extraction, the residual material after re-humidifying will yield a smoke containing the same compounds albeit in smaller amount than would be produced from the unextracted tobacco. The conclusion is unavoidable that the hydrocarbons are produced from the constituent materials of the tobacco during the smoking process. The major constituents of tobacco for use by smokers are well known and are fully discussed in the standard works on tobacco technology (e.g. Garner, 1946 ; Frankenburg, 1946). It was considered appropriate to examine these substances by experimental means to determine which of them gives rise to such hydrocarbons during the smoking process and the object of the present paper is to present the results of the thermal decomposition of some of the major tobacco constituents as a contribution towards this end. In Table I are listed these major constituents for a typical flue-cured cigarette tobacco with their approximate proportions expressed as percentages of dry weight (Frankenburg, 1946). The quantity of each constituent is somewhat variable and depends upon many factors such as the variety of tobacco plant, the soil composition, the weather conditions during growth, the time of harvesting and the rapidity and method of curing.

TABLE I.—*Composition of Flue-cured Cigarette Tobacco*

| | | | |
|---|---|---|---|
| Cellulose | 9·0 | Oxalic acid | 1·0 |
| Lignin | 3·5 | Citric acid | 0·6 |
| Pectins | 10·7 | Total nitrogen compounds | 11·1 |
| Starch | 4·0 | | |
| Sucrose | 4·2 | Total resins | 9·8 |
| Glucose | 11·0 | Polyphenols | 2·0 |
| Fructose | 7·8 | Hemicelluloses | 1·6 |
| Malic acid | 10·1 | Ash | 13·2 |
| | | Total | 99·6 |

One of the major constituents, cellulose (in the form of cigarette paper) has been smoked in a manner as closely resembling cigarette smoking as was possible

(Cooper, Gilbert and Lindsey, 1955) and the presence of polycyclic aromatic hydrocarbons has been demonstrated in the smoke. This has been confirmed by Cardon, Alvord, Rand and Hitchcock (1956), and by Latarjet, Cusin, Hubert-Harbart, Muel and Royer (1956). It was decided that, since thermal decomposition must be one of the processes occurring in the smoking process, some useful information should be obtained by heating each of the tobacco constituents to the prevailing temperature during the production of smoke. Accordingly the first ten substances in Table I of the purest form obtainable were heated for one hour at 650° C. in an atmosphere of nitrogen, and the products of the thermal decomposition were collected and examined by the methods of alumina chromatography and ultra-violet spectrophotometry employed in the examination of tobacco smoke.

### EXPERIMENTAL

The pure chemical compounds employed were of Analar grade. The specimen of cellulose used was the highest grade of absorbent cotton wool, the lignin was used as supplied from the Forest Products Research Laboratory and was obtained from the sulphite liquors of the wood pulp industry, the pectin was manufactured by Unipektin Ltd., of Zurich, and the starch was washed wheat starch.

All the glass apparatus employed was vigorously cleaned by the techniques previously described and care was taken throughout the investigation to minimize exposure of the materials or products to atmospheric pollution. The reagents employed were purified as previously described.

A weighed quantity (usually 5 g.) of the material to be examined was placed in a pyrex glass tube through which a slow stream of nitrogen was passed. When all the air had been displaced, an electric furnace previously adjusted to 650° C. was rapidly raised to surround the tube and the products of thermal decomposition were collected in a water-cooled condenser and receiver. All connections were made by means of standard ground-glass joints. Heating was continued for one hour and the tube was then allowed to cool in the nitrogen stream. The whole apparatus including the carbonized residue was extracted with acetone and the acetone solution transferred to cyclohexane as in the tobacco smoke analysis. After washing with acid and sodium hydroxide, the neutral cyclohexane solutions were analysed on activated alumina columns 10 cm. long and 1·2 cm. diameter. Good separations of the hydrocarbons were usually obtained with one chromatogram. The quantities of each polycyclic hydrocarbon determined are shown in Table II expressed in micrograms for 100 g. of the material used.

### DISCUSSION

The smoking process is a complicated series of changes as yet imperfectly understood. The complications are enhanced by the variable nature of the initial material and by the additional variables introduced by the smoking technique. It is possible however to formulate some fundamental processes that must occur in all smoking and to discuss in qualitative terms the effect of such processes upon the products.

All smoking involves the smouldering (slow, incomplete combustion at a relatively low temperature) of the material aided by intermittent currents of air drawn through by the smoker. The smoke drawn in by the smoker during

TABLE II.—*Polycyclic Hydrocarbons from Tobacco Constituents (in micrograms per 100 grams at 650° C.)*

| | Cellulose | Lignin | Pectin | Starch | Sucrose | Glucose | Fructose | Malic acid | Citric acid | Oxalic acid |
|---|---|---|---|---|---|---|---|---|---|---|
| Acenaphthylene . | 160 | 80 | 20 | 56 | 24 | 27 | 104 | 16 | 50 | 15 |
| Fluorene . . | 584 | 8000 | 287 | 32 | 12 | 7 | 118 | 632 | 173 | 3 |
| Anthracene . | 337 | 544 | 539 | 104 | 70 | 36 | 139 | 70 | 98 | 30 |
| Pyrene . . | 219 | 33 | 133 | 35 | 24 | 66 | 35 | 166 | 24 | 2 |
| Fluoranthene . | 164 | 58 | 152 | 94 | 35 | 45 | 1C6 | 136 | 6 | — |
| 3-Methylpyrene * | 131 | — | 29 | 11 | 15 | 1 | 11 | 119 | 89 | 1 |
| 1 : 2-Benzanthracene | 186 | 44 | 273 | 116 | 41 | 43 | 120 | 130 | 5 | — |
| 1 : 2-Benzpyrene . | 65 | 22 | 34 | 4 | 2 | 11 | 2 | 8 | 37 | — |
| 3 : 4Benzpyrene . | 78 | 47 | 45 | 17 | 10 | 29 | 33 | 35 | 17 | 1 |
| Anthanthrene . | 10 | — | 9 | 1 | — | 5 | 5 | 1 | 2 | 0·3 |
| Coronene . . | 44 | — | 15 | 3 | 3 | 1 | 5 | 6 | 11 | 2 |

*Notes :*

* This compound may be another alkyl pyrene.

The compounds azulene, naphthalene, an alkyl naphthalene, acenaphthene, phenanthrene and perylene were also found and determined in some of the products.

these periods (the main-stream smoke) is the material upon which most interest is focussed. The side stream smoke rising from the hot material during quiescent periods has been neglected by most investigators. The fundamental processes involved are combustion, distillation, steam distillation, thermal decomposition and its sequences.

(i) Combustion of organic material is most complete at elevated temperatures. It is only partial in smoking (low temperatures) conditions ; that is, unused oxygen from the air passes into the mainstream smoke and carbon monoxide also is present as well as the complete combustion products carbon dioxide and water. It should be noted that some carbon dioxide originates from decarboxylation reactions.

(ii) Distillation proceeds in the close proximity of the glowing " coal ". The high temperature volatilizes compounds of appropriate boiling point from tobacco adjacent to the coal and many of these re-condense on cooler material a short distance further from the hot zone. The temperature gradient is very steep (Harlow, 1956). Upon suction such compounds will be re-volatilized in hot gases from the air current and will again partially condense. Successive distillations of this kind result in a certain fractionation of the volatile materials. A further aspect of the slow distillation during quiescent periods and rapid volatilization of distilled products on suction is the accumulation of the volatile materials of rather higher molecular weight in the residual tobacco (stubs and dottles).

(iii) Steam distillation is brought about by the volatilization of the moisture of the tobacco as well as by the steam produced as a product of combustion. The current of steam carries volatile compounds into the mainstream smoke with considerable facility, at temperatures below 100° C. Thus substances which in dry conditions would decompose upon heating may be carried unchanged into the mainstream smoke. Distillation and steam distillation inevitably proceed at the same time.

(iv) Thermal decomposition occurs in the smoking process with a very large number of compounds when they become heated to sufficiently high temperatures.

Mostly the less volatile and the almost non-volatile compounds will undergo thermal decomposition and the products will pass into the mainstream smoke. Free radicals are almost certainly formed at this stage and then combine together or with other compounds to produce new compounds. Within this classification must be included reactions that are often called pyrolytic regardless of etymology, that is the synthetic reactions consequent upon true pyrolysis. And it is probably such reactions that give rise to the polycyclic aromatic hydrocarbons.

The first conclusion to be drawn from the experimental results is that all the substances examined (totalling in quantity to 61·9 per cent of an average cigarette tobacco) give rise to considerable quantities of polycyclic hydrocarbons upon thermal decomposition. The amounts, calculated in proportion to the percentage of each constituent would, if produced in the same quantities in smoking, give rise to much greater amounts (of the order of 10 times) than are actually found in smoke. Larger quantities are to be expected because no combustion occurs in the thermal decomposition experiments. It may therefore be concluded that the smoking process is responsible for the reduction of the quantities that are produced in the thermal reactions alone.

This, a plausible conclusion, is in harmony with the findings of Lyons (1955) who showed that 3 : 4-benzpyrene when added to cigarettes is only partially recovered from the smoke. It should be noted that some of the tobacco constituents are destroyed by combustion before they can be decomposed thermally.

The production of these compounds from tobacco constituents is also in harmony with the experiments of Roffo (1939) who showed that the tar obtained by the dry distillation of tobacco contained 3 : 4-benzpyrene and was carcinogenic and also with our own experiments upon tobacco and sawdust of quercus japonica, which when heated to 650° C. gave rise to a series of polycyclic aromatic hydrocarbons. These experiments also recall those of Kennaway (1924, 1925) in which many organic substances heated to a high temperature produced carcinogenic tars.

One may draw the simple general conclusions that organic substances usually give polycyclic aromatic hydrocarbons when heated to red heat and that if combustion processes are partial only, these compounds will persist in the product. They are inevitable constituents of smokes.

Other pyrolytic processes which have been described recently should be mentioned in connection with this discussion. The paraffin hydrocarbons which are well known constituents of tobacco have been heated to 800° C. (Lam, 1955) who recognized 3 : 4-benzpyrene, 1 : 2-benzpyrene, naphthalene, pyrene and anthracene in the products. A considerable number of low molecular weight compounds have been shown to be present in the gaseous phase of cigarette smoke (Adamek, Hobbs and Osborne, 1956) and of these several, e.g. methane, ethylene and acetylene, are known to polymerize readily to give polycyclic hydrocarbons, of which they could well be the precursors, in the smoking process.

### SUMMARY

A number of the major constituents of cured tobacco has been subjected to thermal decomposition at 650° C. in the absence of air. In the neutral fraction of the decomposition products polycyclic aromatic hydrocarbons have been detected and determined by the methods already developed for the analysis of tobacco smoke. The quantities are much greater than are found in the smoke from equivalent quantities of tobacco. It is concluded that thermal decomposition is in large measure responsible for the presence of these compounds in tobacco smoke and that, since they originate from major constituents of the tobacco, they are likely to be present in all smoke whatever pre-treatment is given to the tobacco.

The authors acknowledge the provision of chemicals by Imperial Chemical Industries Ltd. and gifts of specimens of lignin from Dr. F. Y. Henderson of the Forest Products Research Laboratory, Department of Scientific and Industrial Research, and pectin from Dr. H. G. Harvey of the Food Manufacturers Research Association. They also thank the Medical Research Council for supporting the investigation and Professor Sir Ernest Kennaway, for his interest and helpful criticism.

### REFERENCES

ADAMEK, S., HOBBS, M. E. AND OSBORNE, J. S.—(1956) *Analyt. Chem.*, **28**, 211.

BONNET, J. AND NEUKOMM, S.—(1956) *Helv. chim. Acta*, **39**, 1724.

CAMPBELL, J. M. AND LINDSEY, A. J.—(1956) *Brit. J. Cancer*, **10**, 649.—(1957) *Ibid.*, **11**, 192.

CARDON, S. Z., ALVORD, E. T., RAND, H. J. AND HITCHCOCK, R.—(1956) *Ibid.*, **10**, 485.

COMMINS, B. T., COOPER, R. L. AND LINDSEY, A. J.—(1954) *Ibid.*, **8**, 296.

COOPER, R. L., GILBERT, J. A. S. AND LINDSEY, A. J.—(1955) *Ibid.*, **9**, 442.

*Idem* AND LINDSEY, A. J.—(1953) *Chem. & Ind.* (*Rev.*), 1205.—(1954) *Ibid.*, 1260.

*Idem*, LINDSEY, A. J. AND WALLER, R. E.—(1954) *Ibid.*, 1418.

FRANKENBURG, W. G.—(1946) 'Advances in Enzymology'. New York (Interscience Publishers Inc.).

GARNER, W. W.—(1946) 'The Production of Tobacco'. Philadelphia (Blakiston).

GILBERT, J. A. S. AND LINDSEY, A. J.—(1956) *Brit. J. Cancer*, **10**, 642, 646.

HARLOW, E. S.—(1956) *Science*, **123**, 226.

KENNAWAY, E. L.—(1924) *J. Path. Bact.*, **27**, 238.—(1925) *Brit. med. J.*, ii, 3.

LAM, J.—(1955) *Acta path. microbiol. scand.*, **37**, 421.

LATARJET, R., CUSIN, L. J., HUBERT-HARBART, M., MUEL, B. AND ROYER, R.—(1956) *Bull. Ass. franç. Cancer*, **43**, 180.

LETTRÉ, H. AND JAHN, A.—(1955) *Naturwissenschaften*, **42**, 210.

*Iidem* AND HAUSBECH, C.—(1956) *Z. angew. Chem.*, **68**, 212.

LYONS, M. J.—(1955) *Rep. Brit. Emp. Cancer Campgn*, **33**, 278.

ROFFO, A. H.—(1939) *Z. Krebsforsch.*, **49**, 588.

SEELKOPF, C.—(1955) *Z. LebensmittUntersuch.*, **100**, 218.